# Licraside as novel potent FXR agonist for relieving cholestasis: structure-based drug discovery and biological evaluation studies

**DOI:** 10.3389/fphar.2023.1197856

**Published:** 2023-06-15

**Authors:** Lili Xi, Axi Shi, Tiantian Shen, Guoxu Wang, Yuhui Wei, Jingjing Guo

**Affiliations:** ^1^ Office of Institution of Drug Clinical Trial, The First Hospital of Lanzhou University, Lanzhou, China; ^2^ Department of Pharmacy, The First Hospital of Lanzhou University, Lanzhou, China; ^3^ The First School of Clinical Medicine, Lanzhou University, Lanzhou, China; ^4^ School of Pharmacy, Lanzhou University, Lanzhou, China; ^5^ Centre in Artificial Intelligence Driven Drug Discovery, Faculty of Applied Sciences, Macao Polytechnic University, Macao, China

**Keywords:** FXR, licraside, agonist, cholestasis, virtual screening, biological evaluation

## Abstract

Cholestasis is a common clinical disease caused by a disorder in bile acids (BAs) homeostasis, which promotes its development. The Farnesoid X receptor (FXR) plays a critical role in regulating BAs homeostasis, making it an essential target for cholestasis treatment. Although several active FXR agonists have been identified, effective drugs for cholestasis are still lacking. To address this, a molecular docking-based virtual screening method was used to identify potential FXR agonists. A hierarchical screening strategy was employed to improve the screening accuracy, and six compounds were selected for further evaluation. Dual-luciferase reporter gene assay was used to demonstrate FXR activation by the screened compounds, and their cytotoxicity was then evaluated. Among the compounds, licraside showed the best performance and was selected for *in vivo* evaluation using an ANIT-induced cholestasis animal model. Results demonstrated that licraside significantly reduced biliary TBA, serum ALT, AST, GGT, ALP, TBIL, and TBA levels. Liver histopathological analysis showed that licraside also had a therapeutic effect on ANIT-induced liver injury. Overall, these findings suggest that licraside is an FXR agonist with potential therapeutic effects on cholestasis. This study provides valuable insights into the development of novel lead compounds from traditional Chinese medicine for cholestasis treatment.

## 1 Introduction

Cholestasis is a pathological condition that disrupts bile formation, excretion, and enterohepatic circulation, leading to the retention of toxic bile constituents, including bile salts (Ghonem et al., 2015). Bile acids (BAs) are endogenous substances that play critical physiological roles, including absorption and transport of fat and fat-soluble vitamins, elimination of cholesterol, and regulation of gut microbiome. More importantly, BAs serve as signaling molecules that activate the farnesoid X receptor (FXR), regulating their own synthesis, transport, and glucose and lipid homeostasis. Disruption of this balance can cause cholestasis, which if untreated, can progress to hepatitis, hepatic fibrosis, cirrhosis, and liver tumors (Maillette de Buy Wenniger et al., 2010; Zhu et al., 2016; Cruz-Ramón et al., 2017). Therefore, regulation of BAs pathways has become a novel strategy to treat cholestasis. FXR (NR1H4) belongs to the superfamily of nuclear receptors (NRs) and is involved in the regulation of BAs homeostasis by controlling their synthesis, transport, and metabolism (Keitel et al., 2019). Activation of FXR by agonists can directly activate the transcription of genes encoding small heterodimer partner (SHP) in the liver, which represses the expression of CYP7A1 and CYP8B1 to reduce the synthesis of BAs (Kong et al., 2012), then down-regulates the expression of Na^+^-dependent taurocholic acid cotransporter (NTCP) to reduce hepatic uptake of BAs, and increases the expression of multidrug resistance associated protein 2/3 (MRP2/3) and bile salt export pump (BSEP) to promote the secretion of BAs from liver to bile. Moreover, FXR can induce phase I and II reactions to increase the hydrophilicity of BAs, making it easier to be excreted through urine. Studies in animal models have found that targeted deletion of Fxr results in increased levels of serum bile acids, while mice with FXR gene knockout show BAs accumulation and liver injury (Sinal et al., 2000; Kong et al., 2019). Genetic variants in human FXR are associated with a spectrum of cholestatic disorders (Van Mil et al., 2007; Gomez-Ospina et al., 2016). In conclusion, FXR plays an important role in the pathogenesis of cholestasis, and specific activation of FXR represents a promising way to treat cholestasis.

FXR has emerged as a potential therapeutic target for cholestatic liver disorders (Stofan et al., 2020), and its agonists have gained approval for the treatment of primary biliary cholangitis (PBC) and primary sclerosing cholangitis (PSC). However, the use of the FXR agonist obeticholic acid (OCA) has resulted in several side effects, including pruritus and an increased risk of hepatic decompensation in cirrhotic PBC patients (Nevens et al., 2016). Despite this, a large number of clinical and preclinical studies are still seeking novel and effective FXR agonists for the treatment of cholestasis and related diseases. Natural products are a promising source for bioactive agents and lead compounds for drug discovery (Hiebl et al., 2018; Wang et al., 2020). Recently, Picroside II and Yangonin were reported to exert protective effects against cholestasis through FXR activation (Gao et al., 2018; Li et al., 2020), and 72 natural compounds were identified as FXR agonists (She et al., 2021). However, many lead compounds have failed in preclinical and clinical developments due to toxicological and/or pharmacokinetic issues, and further discovery of FXR agonists from natural resources remains of great value. FXR has typical structural features of the NR family and binds to the retinoid X receptor (RXR) in the form of heterodimers to regulate transcription of downstream genes (Sever et al., 2013; Wang et al., 2018; Zheng et al., 2018). The ligand binding domain (LBD) of FXR is composed of 12 *a*-helices and induces a conformational change in the activation domain (AF2) (Jin et al., 2010; Ding et al., 2015) upon binding appropriate ligands, leading to the release of co-repressors and activation of coactivators to regulate target gene transcription.

This study utilized a hierarchical screening strategy that combined *in silico*, *in vitro*, and *in vivo* methods to identify natural FXR agonists. Molecular docking, using the Glide program (Sastry et al., 2013), was employed to screen for potential FXR/RXR heterodimer targeting compounds from natural products and monomer compounds of traditional Chinese medicine. Six compounds were selected for toxicity and activation activity testing through MTT assay and dual-luciferase reporter gene assay *in vitro*. One hit compound, licraside, was chosen for further validation through animal experiments, which included biochemical and liver histological analysis to assess its potential therapeutic effect on cholestasis. Overall, this strategy provided a rapid and effective approach to discovering novel FXR agonists from natural sources.

## 2 Materials and methods

### 2.1 Protein preparation and grid generation

The 3D structure of human FXR-LBD complexed with ligand WAY-362450 was retrieved from Protein Data Bank (PDB code: 5Z12) and prepared using the Protein Preparation Wizard (Epik, 2021; Impact, 2021; Prime, 2021). This involved adding hydrogens, assigning charges using OPLS_2005 force field, protonation states at pH 7 ± 2, and energy minimization using Impact Refinement module. The ligand was removed prior to grid generation, and the Receptor Grid Generation module (Glide, 2021) was used to calculate the binding site and generate the grid files for docking in Glide program. The co-crystalized ligand was used as the centroid for grid generation with default settings and a scaling factor of 1.0 for protein van der Waals radii.

### 2.2 Dataset: source and preparation

In a total of 121,341 compounds retrieved from Traditional Chinese Medicine Systems Pharmacology Database (TCMSP) and Traditional Chinese Medicine Natural Product library (TCMNP) (Chen, 2011; Ru et al., 2014), were used for virtual screening in this work. Energy minimization was performed by Impact module, using OPLS_2005 force field with a distance-dependent dielectric and conjugate gradient algorithm, and others were default values. All the optimized compounds were prepared with LigPrep module (LigPrep, 2021) before docking, and all species existing at pH = 7 ± 2, including tautomers and enantiomers, were generated. Using OPLS_2005 force field in vacuum, a short conformational search was performed to relax the structures.

### 2.3 Evaluation of molecular docking and virtual screening

The Glide module was evaluated for docking ability and reliability by re-docking the co-crystallized ligand WAY-362450 into the active sites of FXR using standard precision (SP) and extra precision (XP). The pose with the best XP docking score was selected and compared with the original crystal pose using RMSD values focusing on all heavy atoms of the ligand.

Subsequently, a hierarchical screening strategy was implemented for docking preprocessed compounds into the binding pocket of FXR using Glide. The strategy included HTVS, SP, and XP modes with a van der Waals radii scaling of 0.8 Å and partial charge of 0.15. The top 10% of compounds with the highest docking scores were selected for further studies.

### 2.4 Chemical and reagents

The compounds tested, with a purity of >98%, were purchased from Yuanye Bio-Technology Co., Ltd. (Shanghai, China). Additionally, *a*-naphthylisothiocyanate (ANIT), OCA, and CDCA with a purity of >98% were purchased from Sigma-Aldrich Chemical Co., Ltd. (Darmstadt, Germany). The dual-luciferase reporter assay kit was procured from Promega (Madison, United States), while the ALT Kit, AST Kit, ALP Kit, and TBA Kit were obtained from Nanjing Jiancheng Institute of Biotechnology (Nanjing, China). Furthermore, the RNAprep Pure Tissue Kit and FastQuant RT Kit were purchased from TIANGEN Biotech Co., Ltd. (Beijing, China), and the FastStart Essential DNA Green Master Kit was acquired from Roche.

### 2.5 Cell culture

HepG2 cells, obtained from the American Type Collection (ATCC), were cultured in MEM medium with 10% FBS and 1% Penicillin/Streptomycin. Standard culture conditions were maintained at 37°C with 5% CO_2_ for *in vitro* experiments.

### 2.6 Hepatotoxicity assay

HepG2 cells were seeded in 96-well plates at a density of 2 × 10^4^ cells/well and allowed to adhere in complete medium under normoxic conditions. The cells were treated with varying concentrations of the tested compounds for 24 or 72 h. Following treatment, MTT (5 mg/ml) was added to each well and incubated for 4 h. The supernatant was then removed, and 200 µL DMSO was added to each well. Finally, absorbance was measured at 490 nm using a microplate reader.

### 2.7 Plasmid construction, transient transfection and dual-luciferase reporter gene assays

FXR expression plasmid was constructed by cloning genes encoding NR1H4 into GV219 mammalian expression vector. The plasmid was constructed by Shanghai Genechem. BSEP promoter reporter was constructed by cloning a genomic DNA fragment into the luciferase vector GV238. For FXR agonistic activity assay, HepG2 cells (1.5 × 10^4^ cells/well) were transiently transfected with FXR expression plasmid (1 µg), BSEP promoter luciferase reporter vector (1 µg) and the null-Renilla luciferase plasmid (0.1 µg) as an internal control for 24 h. Cells were treated with vehicle (0.1% DMSO in medium), positive control CDCA (10 µM), and series of concentrations of the tested compounds (20 μM, 40 μM, 80 µM). After 24 h, the luciferase activities were determined by a dual-luciferase reporter gene assay system. Dual-luciferase reporter assay kit was preformed to measure luciferase activities according to the manual instruction. The final luminescence was normalized based on the Renilla luminescene signal, and the ratio of treatment over control served as-fold activation. Data were represented as means ± standard deviations (SD.) of three individual transfections.

### 2.8 Western blot analysis

After 24-h treatment with licraside at concentrations of 40 µM and 80 μM, as well as 10 µM CDCA, HepG2 cells were subjected to protein extraction. Total protein was extracted from the cells using ice-cold RIPA buffer (Solarbio, R0010, Beijing, China).

Equivalent protein samples were then separated using SDS-PAGE and transferred to polyvinylidene difluoride membranes (Immobilon-P, IPVH00010, United States). Following blocking with 5% non-fat milk, the membranes were incubated overnight at 4°C with rabbit anti-BSEP (Abcam, ab155421, Cambridge, England) and rabbit anti-SHP (Origene, TA379281, Jiangsu, China) antibodies. Subsequently, the membranes were incubated with the appropriate secondary antibody conjugated with HRP at room temperature for 1 h. Membranes were incubated for 20 min with chemiluminescent HRP substrate (Immobilon Western, WBKLS0100, United States) and collected with the gel imagine system (Tanon, Shanghai, China). The relative expression of proteins was quantified using ImageJ software (Wayne Rasband, NIH, United States).

### 2.9 Animals, treatments and sample collection

Male C57BL/6 J mice (8–10 weeks, 18–20 g) were obtained from the Veterinary Institute, Chinese Academy of Agricultural Sciences (Lanzhou, China). Animals were housed in an air-conditioned room (25°C ± 1°C) under a 12-h light/dark cycle for 1 week acclimatization and allowed water and standard rodent chow *ad libitum*. Furthermore, all efforts were made to minimize the number and suffering of animals used.

Mice were randomly divided into six groups (*n* = 8 in each group), and the administration protocol of the animal experiments was shown in Figure 5A: 1) control group (the same volume of vehicle), 2) ANIT group (ANIT 75 mg/kg, in olive oil), 3) low dose group (licraside 1 mg/kg + ANIT, marked as L-group), 4) medium dose group (licraside 5 mg/kg + ANIT, marked as M-group), 5) high dose group (licraside 10 mg/kg + ANIT, marked as H-group), 6) positive drug group (OCA 10 mg/kg + ANIT). Licraside or vehicle was injected intraperitoneally once a day for 7 days. OCA was administered to mice by oral gavage once a day for 7 days. Since the fifth day, mice were orally administered 75 mg/kg dose of ANIT in olive oil.

On the seventh day, 2 h after administration, blood of eyeball and liver tissue were collected after animals were euthanized with an overdose of anesthesia. Serum was collected by centrifugation for biochemical index determination. Liver tissue samples were collected and quickly frozen in liquid nitrogen at −80°C for liver histological HE staining.

### 2.10 Biochemical indexes detection

The contents of alanine aminotransferase (ALT), aspartate amino transferase (AST), alkaline phosphatase (ALP), total bilirubin (TBIL) and total bile acids (TBA) in serum obtained after centrifugation were determined by Chemistry Analyzer (OLYMPUS AU400, Tokyo, Japan). TBAs level in serum were measured by using Kit from Nanjing Jiancheng Bioengineering institute (Nanjing, China) and detected by a Chemistry Analyzer (OLYMPUS AU400, Tokyo, Japan), according to the manufacturer’s instructions. The whole gallbladder was removed and placed in a test tube to collect bile, bile samples were diluted to 40-fold with normal saline, and TBA content was measured with TBA Kit (Nanjing Jiancheng Bioengineering institute, Nanjing, China). The results were the mean value of eight different animals.

### 2.11 Histopathology

Liver tissue (*n* = 5) were collected and fixed in 4% formaldehyde for more than 24 h and then embedded in paraffin. The paraffin-embedded tissue was cut into 5 μm thick sections, and hematoxylin and eosin staining were carried out. A pathologist in the pathology department of the First Hospital of Lanzhou University examined pathological sections under a traditional microscope for blinded histopathological evaluation.

### 2.12 Statistical analysis

All experimental data are reported as arithmetic means ± S.D. For statistical analysis, two-tailed Student’s *t*-test was used to deal with variance analysis of unpaired samples and *p* values < 0.05 and < 0.01 were considered statistically significant and very significantly different, respectively.

## 3 Results

### 3.1 Docking method verification and discovery of hit compounds by VS.

**FIGURE 1:**
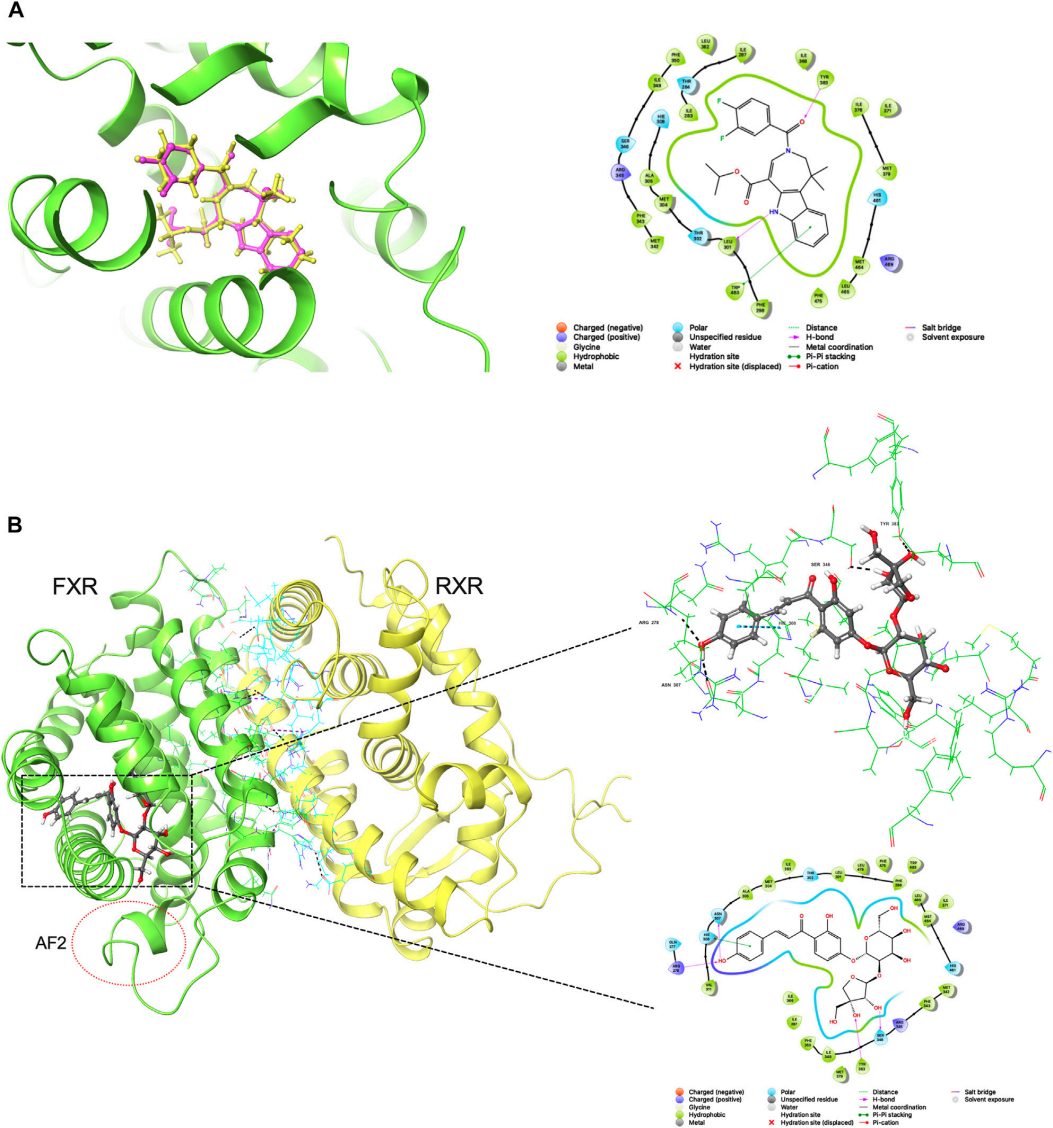
**(A)** Redocking analysis: the superposition of the docked and the co-crystalized poses (33Y in PDB file). Violet: the co-crystalized pose; yellow: the docked pose **(B)** Binding mode of compound 2 in active sites (residues within 3 Å and 2D-interaction was shown).

**FIGURE 2:**
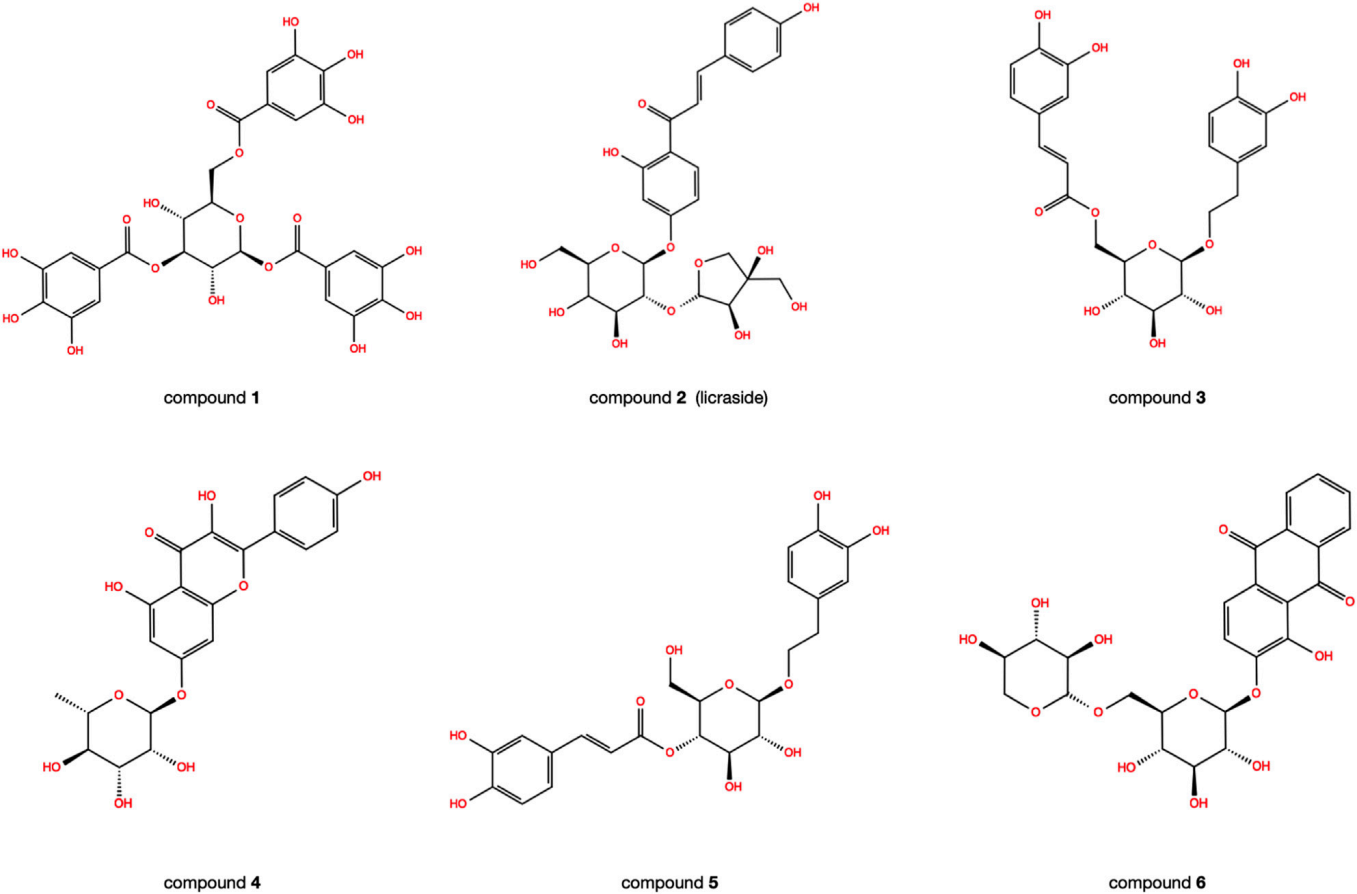
The 2D structures of six candidate compounds.

The reliability of the docking was verified by re-docking the co-crystallized ligand WAY-362450 into active sites of FXR using the Glide program. As shown in Figure 1A, The native pose was accurately reproduced with an RMSD of 0.931 Å, indicating that the native pose could be successfully reproduced by the docking protocol used in this study. An integrated structure-based screening workflow was conducted on more than 120,000 molecular compounds that were firstly pre-filtered by Linpinski’s Rules. The remained molecular compounds were successively screened by the docking protocol of HTVS→SP→XP, with the retain ratio of top 10%, 10% and 10%, respectively. 740 compounds were obtained based on their XP docking score ranking. With consideration of key interactions observed from crystal structures, such as critical hydrophobic interaction and some predominant hydrogen bonds with polar residues, the docked pose of these compounds were retrieved to visual inspection carefully. Six compounds were shortlisted as potential candidates (Figure 2), taking into account their structural diversity and availability for purchase. These compounds were purchased and subjected to *in vitro* and *in vivo* assays.

### 3.2 Evaluation of hepatotoxicity and determination of activation activity of FXR

**FIGURE 3:**
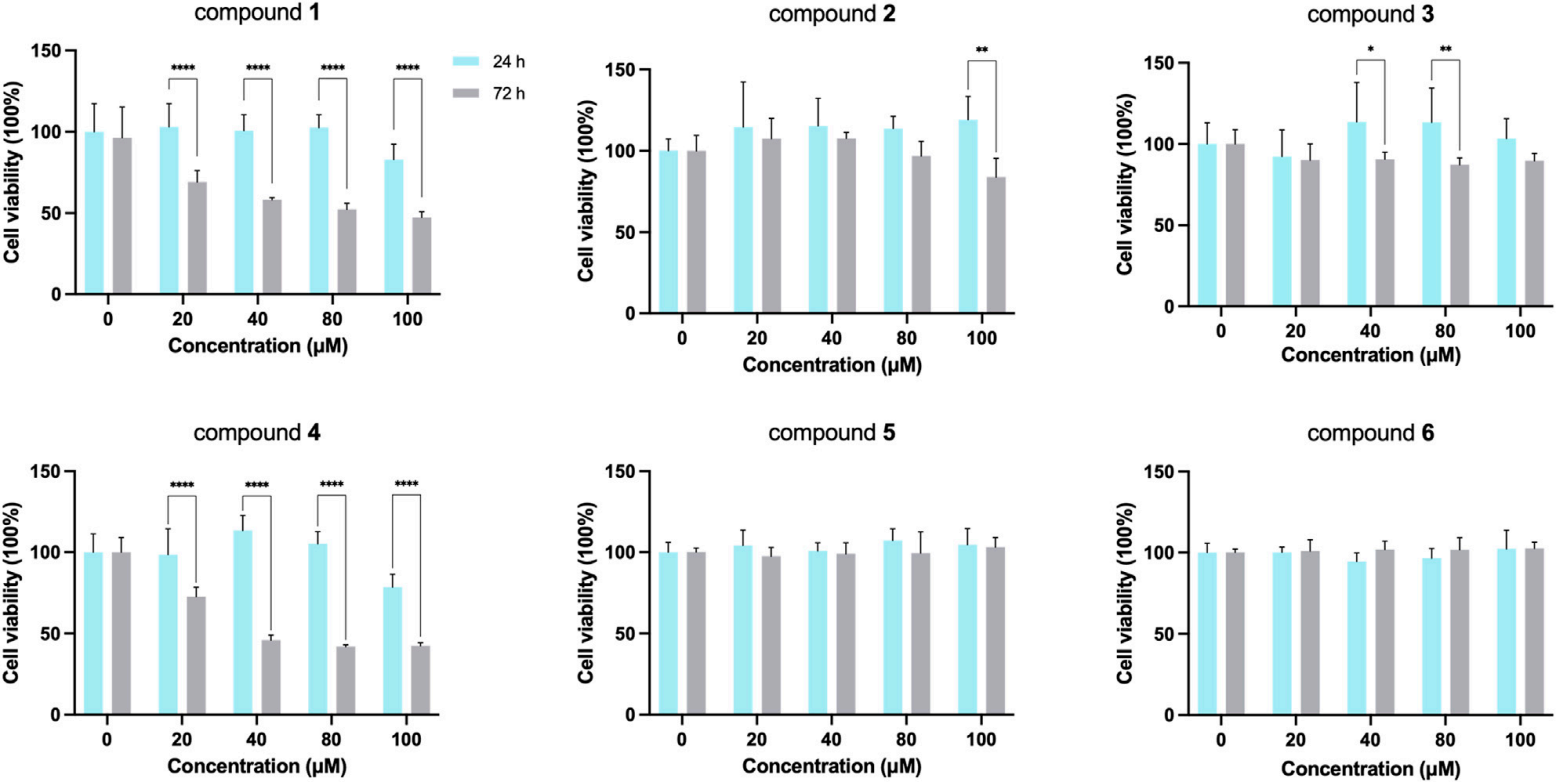
Effect of compounds intervention on cell viability of HepG2 cell (*p < 0.05, **p < 0.01, ***p < 0.005, ****p < 0.001; data is expressed as mean ± SD).

To evaluate the potential hepatotoxicity of six candidate compounds, MTT assay was performed on HepG2 cells, with the purpose of excluding compounds with greater hepatotoxicity and selecting appropriate intervention concentrations for subsequent activity verification experiments. After exposure to various concentrations of 0, 20, 40, 80 and 100 µM for 24 h and 72 h, cell viability was measured. As indicated in Figure 3, there was no significant change in cell status after 24 h intervention with six candidate compounds. Only compounds one and four showed significant hepatotoxicity after 72 h exposure, with the survival rate of HepG2 cells decreasing compared to the control group (*p* < 0.05), and the decrease of cell viability was positively correlated with the concentration. We observed that compound 2 with 20, 40, and 80 μM interventions for 72 h could all result in a slight decrease in cell viability. Non-etheless, this decrease was not significantly different from the effects observed after 24 h of treatment. When reaching 100 µM after 72 h intervention, compound 2 could cause significant hepatic cell death. These findings indicated that prolonged and high-dose interventions may lead to hepatotoxicity. Therefore, a 24 h intervention time was used for subsequent experiments.

**FIGURE 4:**
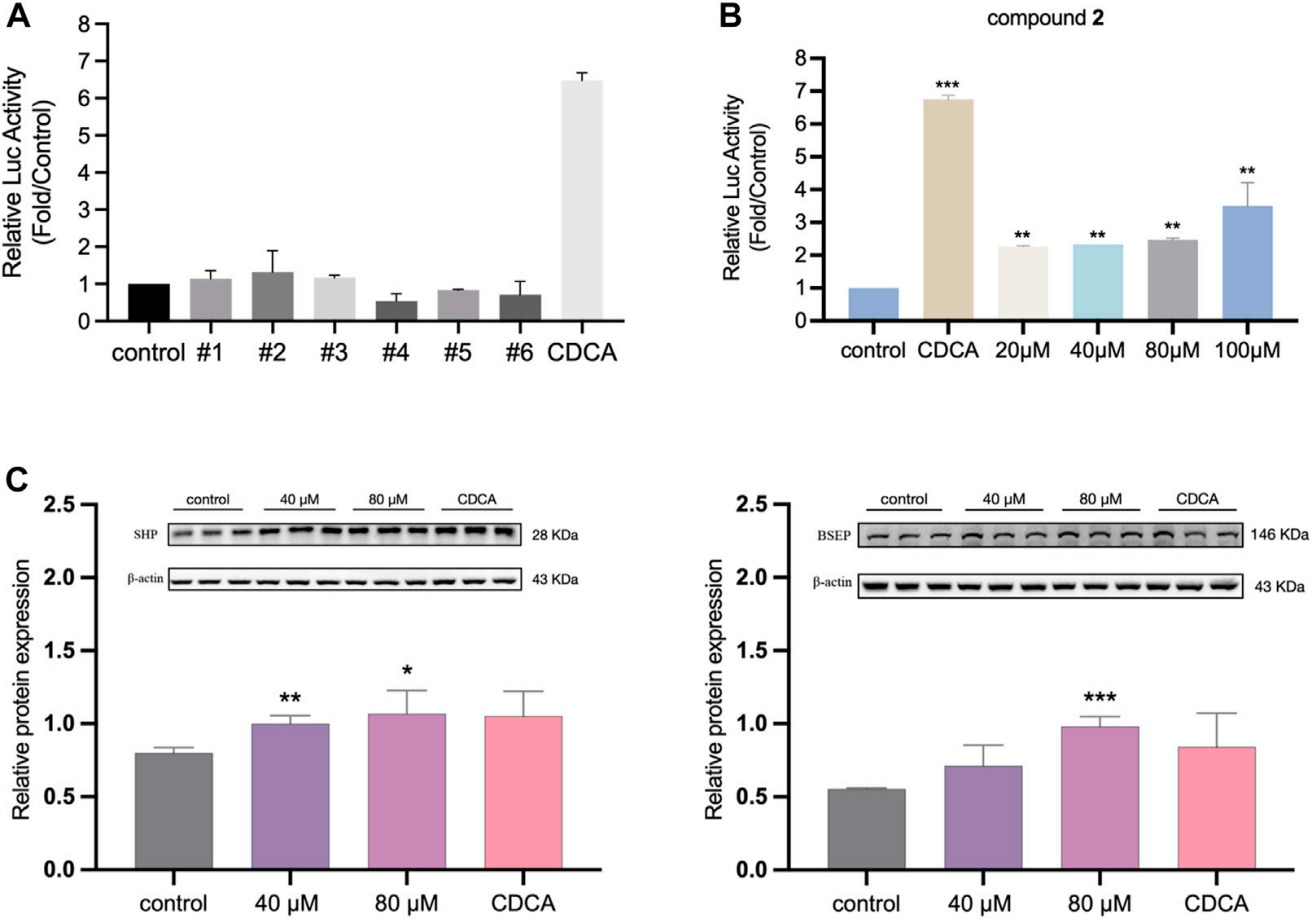
**(A)** The relative luciferase activities of six candidate compounds were evaluated at a single concentration of 20 µM **(B)** The relative luciferase activities of compound 2 in a concentration-dependent manner (*p < 0.05, **p < 0.01, ***p < 0.001 vs control group; data is expressed as mean ± SD.). **(C)** Western blot analysis for FXR, SHP and BSEP expressions after treatment 24 h in HepG2 cells (*n* = 3, data is shown as mean ± SD. *p < 0.05, **p < 0.01, ***p < 0.001 vs. control group.).

The activation activity of the six compounds on FXR was evaluated using a dual-luciferase reporter gene assay. The results indicated that the candidate compounds exhibited weaker activation activity compared to the positive control group (Figure 4A). However, among the compounds tested, compound 2 demonstrated the highest activity and was selected for further concentration-dependent experiments. In Figure 4B, it is illustrated that compound 2 had already effectively activated FXR at the cellular level at the concentration of 20 μM, and the luciferase activity of the reporter gene was significantly increased compared to the vehicle group (*p* < 0.01), albeit not as potent as the endogenous agonist CDCA. Moreover, compound 2 exhibited a dose-dependent activation activity of FXR. When the concentration increased to 100 μM, the activation activity of compound 2 became even stronger. The above results indicated the potency of compound 2 in activating FXR. To further assess its safety, a 24-h MTT assay was conducted, expanding the concentration range of compound 2 up to 800 µM. Supplementary Figure S1 presents the results, indicating that compound 2 did not adversely affect cell viability within the tested concentration range. Even at the highest tested concentration of 800 μM, no decrease in cell viability was observed. Based on these findings, compound 2 (licraside) was selected for subsequent *in vivo* validation due to its potent FXR activation activity and favorable cell viability profile.

### 3.3 Effect of licraside intervention on the expression of FXR, SHP and BSEP in hepG2 cells

To investigate the impact of licraside intervention on the expression of FXR and its downstream signaling proteins in HepG2 cells, Western blot experiment was conducted. The results are presented in Figure 4C. The analysis revealed a significant increase in the level of SHP following licraside intervention compared to the control group (*p* < 0.05). Furthermore, when the intervention concentration of licraside was 80 μM, there was a significant upregulation of BSEP expression (*p* < 0.01). Although BSEP expression also increased in other intervention groups, the differences were not statistically significant. These findings suggested that licraside has the ability to activate FXR and subsequently induce the upregulation of downstream proteins SHP and BSEP *in vitro*.

### 3.4 Body weight changes in ANIT-induced cholestasis model mice

**FIGURE 5:**
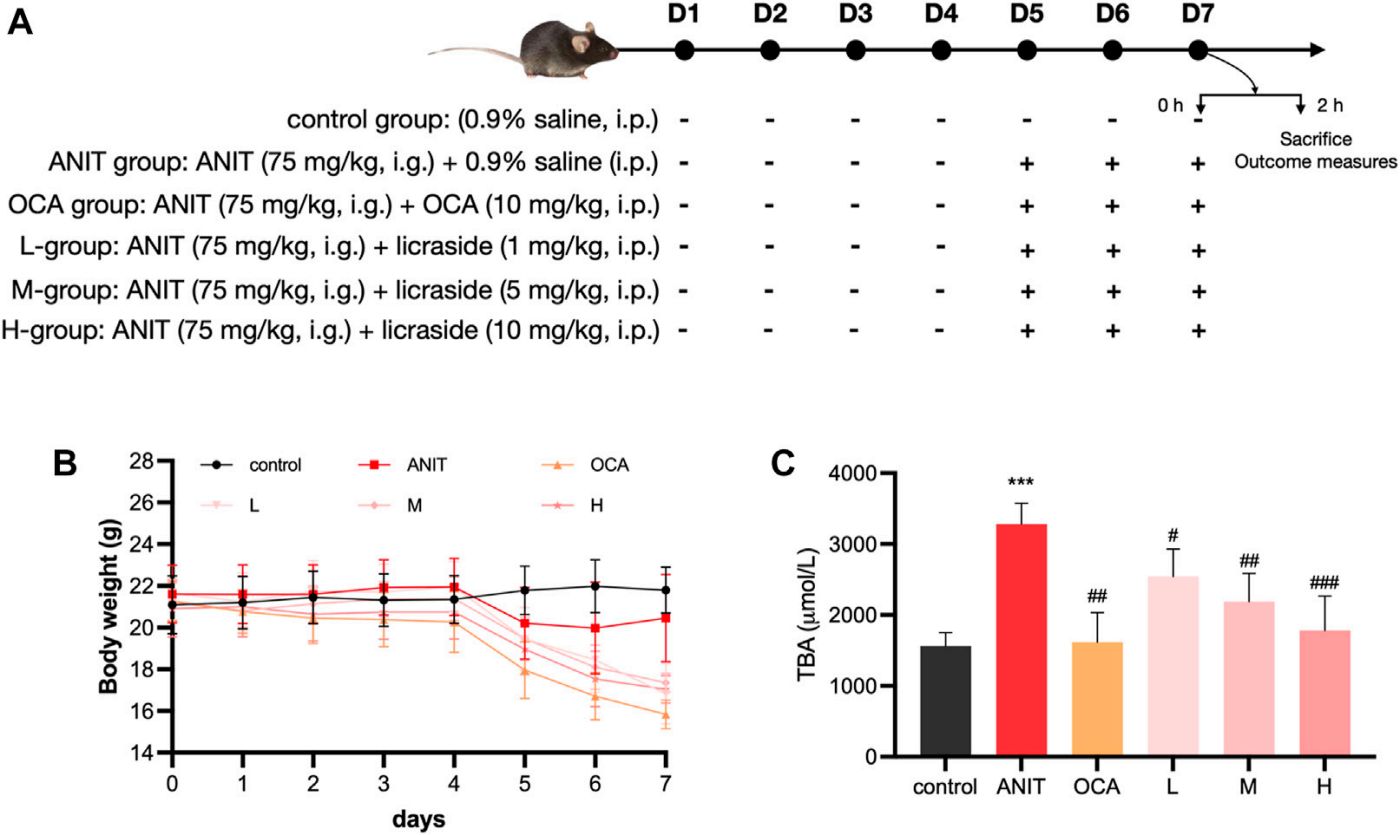
Animal experiment results **(A)** Experimental protocol: the scheme outlining the design and timeline of the animal experiments **(B)** Body weight: the body weight of mice in each experimental group. Data are shown as mean ± SD. with *n* = 8 per group **(C)** TBA level in bile: the levels of TBA in each experimental group (*p < 0.05, **p < 0.01, ***p < 0.001 vs. control group; #*p* < 0.05, ##*p* < 0.01, ###*p* < 0.001 vs. ANIT group, *n* = 8).

The administration protocol for the animal experiment is presented in Figure 5A. Body weight changes were monitored, and the results are illustrated in Figure 5B. During the initial 4 days, minimal fluctuations in body weight were observed across all groups. However, starting from the fifth day, significant weight loss was observed in the ANIT group, the OCA group (positive drug control), and the three licraside groups, compared to the control group. Notably, the OCA group exhibited the most substantial weight loss, consistent with the findings from a phase III clinical trial where OCA treatment in patients led to a dose-dependent decrease in body weight after 18 months (Younossi et al., 2019). These results suggested that the observed weight loss may be attributed to the involvement of FXR in regulating lipid metabolic homeostasis, with alterations in lipid balance contributing to the decrease in body weight.

In the licraside groups, mice in the low- and medium-dose groups experienced slightly less weight loss compared to the ANIT group. Moreover, there was a significant increase in body weight in these groups compared to the OCA group (*p* < 0.05). Importantly, throughout the course of the animal experiments, the mice maintained overall good health. Subsequent pathological analysis revealed significant alleviation of cholestasis symptoms following licraside treatment. Therefore, we concluded that the observed weight loss in mice did not significantly impact the efficacy assessment of licraside in this study.

### 3.5 Effect of licraside on biliary TBA level in ANIT-induced cholestasis model mice

The changes in bile TBA content are shown in Figure 5C. The model group demonstrated a significant increase in TBA levels in bile when compared to the control group (*p* < 0.001), confirming the successful establishment of the intrahepatic cholestasis animal model. Treatment with OCA and licraside at low, medium, and high doses significantly reduced biliary TBA levels (*p* < 0.01). These results indicate that licraside effectively reversed ANIT-induced TBA level increases and that high-dose licraside treatment is comparable to OCA in treating cholestasis.

### 3.6 Effect of licraside on serum biochemical indicators

**FIGURE 6:**
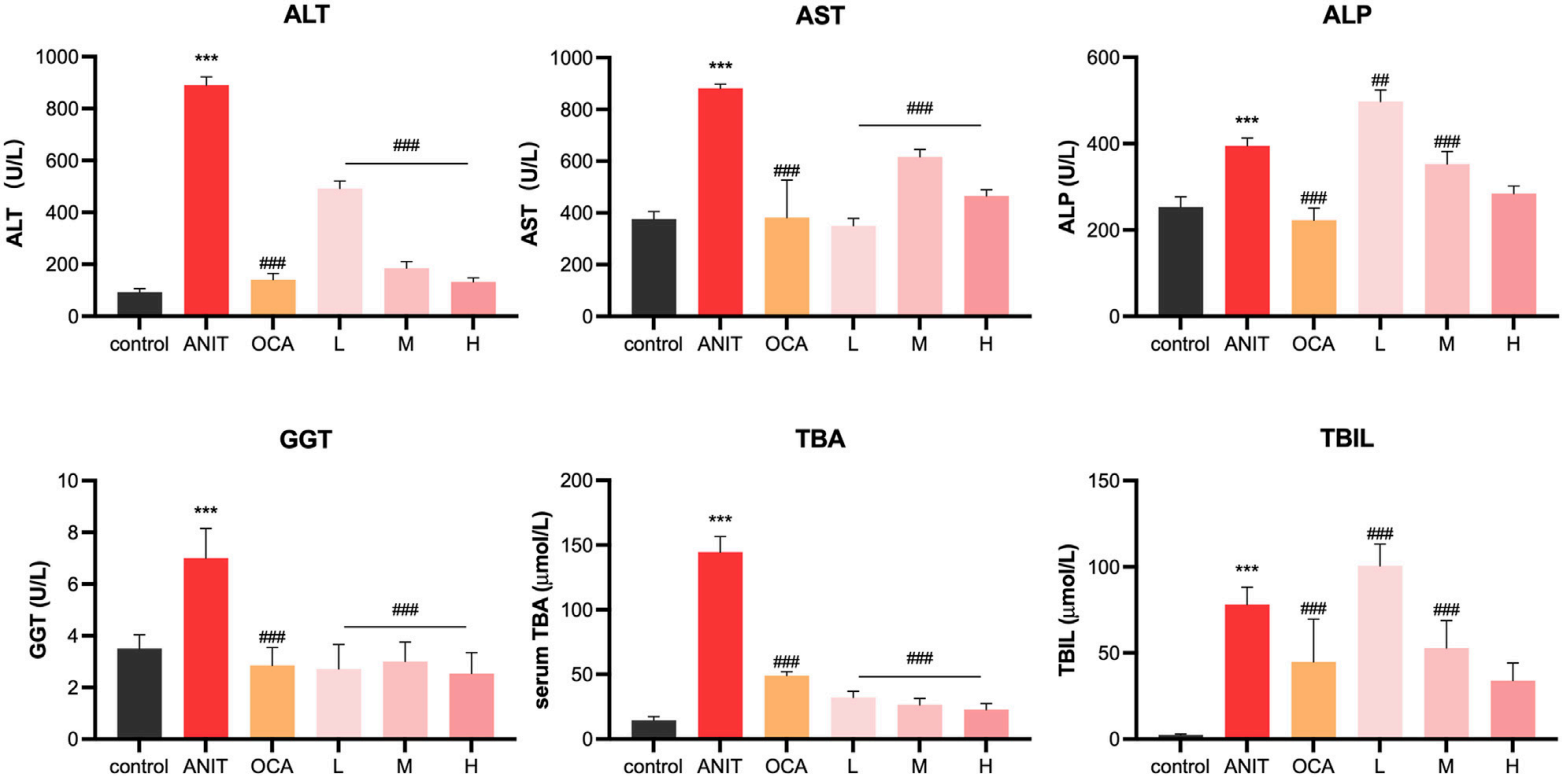
Serum biochemical indicators of ANIT-induced cholestasis animal model (*p < 0.05, **p < 0.01, ***p < 0.001 vs. control group; #*p* < 0.05, ##*p* < 0.01, ###*p* < 0.001 vs. ANIT group, *n* = 3).

ANIT is a hepatotoxic agent used to induce cholestasis in rodents. After ANIT administration, serum ALT and AST levels, markers of liver injury, were markedly elevated, and biomarkers of biliary toxicity, such as ALP, GGT, TBIL, and TBA, were also increased. As shown in Figure 6, compared to the control group, the model group exhibited significantly increased levels of these six biochemical markers in serum (*p* < 0.001), confirming the successful establishment of the intrahepatic cholestasis animal model. Treatment with OCA and licraside at high and medium doses significantly reduced the levels of serum ALT, AST, GGT, ALP, TBIL, and TBA compared to the model group (*p* < 0.001). Consistent with the above conclusions, licraside intervention effectively reversed the elevation of serum biomarkers in the ANIT-induced cholestasis model, with a significant decrease in TBA levels, suggesting the potential efficacy of licraside in relieving cholestasis.

### 3.7 The alleviated effect of licraside on liver pathological changes

**FIGURE 7:**
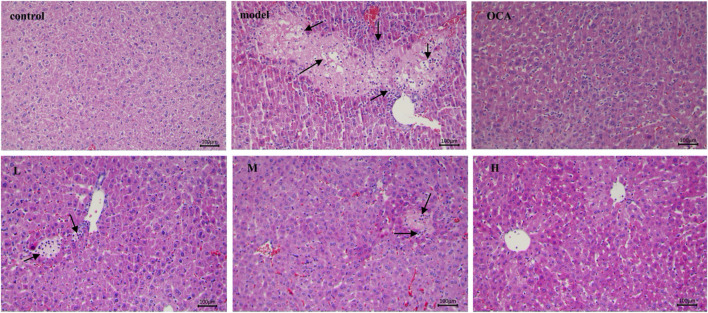
The effect of licraside intervention on the histopathology of liver in ANIT-induced in-trahepatic cholestasis mice (20 ×), control group (control), ANIT group (model), OCA group (OCA), low-, medium- and high-dose licraside groups (L, M, H).

Figure 7 shows representative pathological staining sections of liver tissue. Compared to the control group, ANIT-induced cholestasis model mice exhibited pathological changes such as hepatocyte swelling, diffuse vacuolization, inflammatory cell infiltration, and hepatocyte necrosis, indicating evident hepatocyte damage. After OCA administration, hepatocyte size was consistent, and there was apparent sinusoidal space between them. Low-dose licraside intervention reduced fat vacuole formation and eliminated inflammatory cell infiltration in the bile duct area, with slightly enlarged hepatocytes. Medium-dose licraside intervention showed clear nuclei of liver cells and little hepatocyte necrosis, without fat vacuole formation. High-dose licraside treatment resulted in mice hepatocytes being uniform in size, morphology, and structure, arranged radially and neatly, similar to the control group. These histopathological results indicate that licraside has a significant therapeutic effect on liver injury in ANIT-induced cholestasis mice, and may be useful in alleviating cholestasis.

## 4 Discussion

Symptoms of chronic cholestasis can persist for at least 6 months. While there may be no obvious symptoms in the early stages, hyperbilirubinemia may occur as the disease progresses. If left untreated, bile duct necrosis, inflammation, fibrosis, cirrhosis, and liver failure may occur. BAs disorder promotes the development of cholestasis and is an important pathogenesis. FXR is the main regulator that maintains the homeostasis of BAs, and decreased FXR expression causes abnormal bile acid metabolism (Shearn et al., 2019). Therefore, FXR plays an important role in protecting liver function from the toxic damage of BAs. FXR activation can maintain lipid balance and reduce hepatic steatosis, making it an important therapeutic target in cholestasis. Ursodeoxycholic acid (UDCA) is approved for the treatment of cholestasis, but about 40% of patients with PBC have no respond to UDCA (Santiago et al., 2020). OCA is then approved to treat PBC patients unresponsive to UDCA (Fiorucci et al., 2011; Ali et al., 2016). However, as mentioned earlier, OCA has some side effects. Therefore, the discovery of novel agonists targeting FXR is still of great research value.

In this study, we presented an “*in silico* → *in vitro* → *in vivo*” screening strategy to discover potential novel FXR agonists for the treatment of cholestasis. We used the binding mode of co-crystallized ligand WAY-362450 and FXR for the *in silico* screening process. The binding mode analysis can recognize the important interaction and residues that are responsible for binding affinity. Compound 2 (licraside), which showed better results in subsequent cell experiments, was selected as an example to analyze the binding interaction. The XP Gscore value was −16.192 kJ/mol. The FXR-#2 complex’s conformation (Figure 1B) showed that residues TYR383, SER346, ARG278, and ASN307 were the key players. The phenolic hydroxyl group of licraside acted as both H-bond acceptor and donor, forming two H-bonds with ARG278 and ASN307, and the two hydroxyl groups of the furan ring formed an H-bond with TYR383 and SER346, respectively. The benzene formed a π-π stacking interaction with HIS308. Besides, hydrophobic interaction is also critical. These interactions are similar to that observed in the crystal structure of FXR agonist.

Virtual screening often suffers from a high false positive rate, as discussed by Bender et al. (2021). To mitigate this issue, several strategies have been developed to ensure the reliability of virtual screening, including evaluating docking and screening abilities, analyzing structural diversity, and inspecting binding modes. However, it is important to note that many compounds that appear effective in theory may be inactive *in vitro* and/or *in vivo*, emphasizing the need to sequentially evaluate toxicity and efficacy at both the cellular and animal levels.

To this end, Licraside, an isoglycyrrhizin-4′-O-carinose (1→2) glucoside flavonoid compound derived from licorice, was identified as a potential agonist of FXR that could effectively inhibit TBA and TBIL levels while decreasing ALT, AST, ALP, and GGT levels *in vivo*, thereby demonstrating significant cholagogic effects. Licorice is commonly used in traditional Chinese medicine to treat various ailments, such as gastric, liver, respiratory, and metabolic disorders, and its active extracts possess various medicinal properties, including anti-inflammatory, antioxidative, antimicrobial, antivirus, cell-protective, and chemo-preventive effects.

It is imperative to emphasize that safety considerations hold paramount importance throughout the drug development process. It is duly recognized that the current study has limitations concerning the exploration of toxicity. Therefore, future investigations should incorporate systematic and comprehensive research methods to evaluate the safety profile of licraside thoroughly. Undertaking such measures is essential and imperative to provide robust evidence for further assessment of licraside’s potential as a chemopreventive and therapeutic agent.

Overall, through *in silico* screening, we discovered six hit compounds with clear activation activities targeting FXR, and licraside demonstrated good safety and is a promising lead compound for alleviating cholestasis. This study affirms the feasibility of computer-assisted drug discovery and provides new insights into developing novel agonists for treating cholestasis by targeting FXR.

## Data Availability

The raw data supporting the conclusion of this article will be made available by the authors, without undue reservation.
