# Supplementary figures and images for: Licraside as novel potent FXR agonist for relieving cholestasis: structure-based drug discovery and biological evaluation studies
Source: Front Pharmacol. 2023 Jun 15;14:1197856. doi: 10.3389/fphar.2023.1197856 (PMC10309033; doi:10.3389/fphar.2023.1197856)

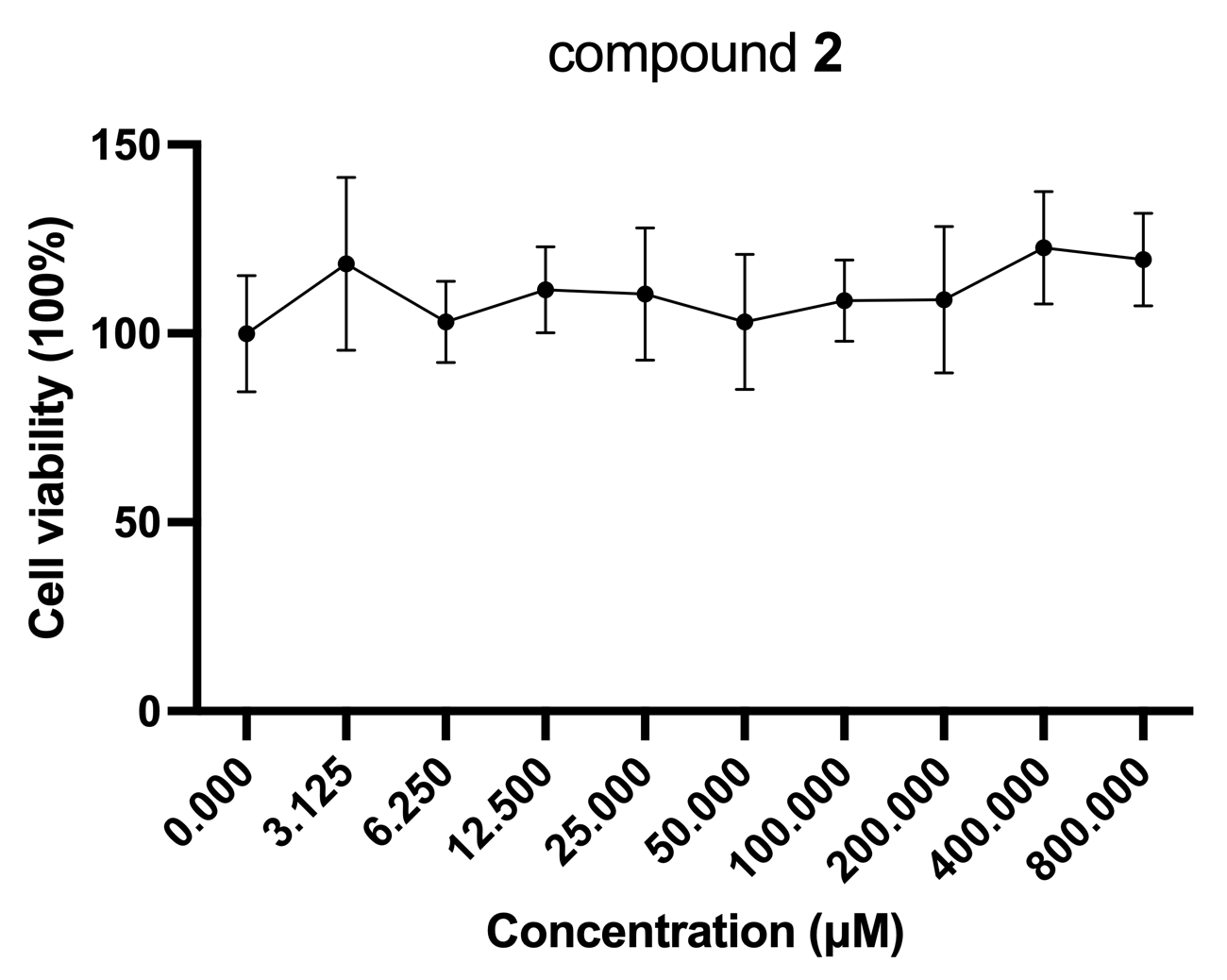

Supplement: Supplementary file 1 [file Image1.TIFF]
